# Using Interactive Family Science Shows to Improve Public Knowledge on Antibiotic Resistance: Does It Work?

**DOI:** 10.1371/journal.pone.0104556

**Published:** 2014-08-27

**Authors:** Donna M. Lecky, Meredith K. D. Hawking, Neville Q. Verlander, Cliodna A. M. McNulty

**Affiliations:** 1 Public Health England Primary Care Unit, Microbiology Department, Gloucester Royal Hospital, Gloucester, United Kingdom; 2 Statistics, Modelling and Economics Department, Public Health England Centre for Infections, London, United Kingdom; Rockefeller University, United States of America

## Abstract

The public plays an important role in controlling the emergence and spread of antibiotic resistance. A large British survey showed that there is still public misunderstanding about microbes and antibiotics. e-Bug, a European DG Sanco sponsored project, aims to disseminate a school antibiotic and hygiene educational pack and website across Europe. Interactive science shows based on the e-Bug educational packs were developed to take the key health and hygiene messages from the e-Bug school resources to families. The science show was evaluated to assess public knowledge and understanding of antibiotics and antibiotic resistance pre and post intervention. An interactive stall comprised of a 3×2 m backing stand with background information, an interactive activity and discussions with a trained demonstrator was on display at a family holiday resort. Pre-piloted knowledge questionnaires were completed by parents and children pre and post intervention. Adult (≥19 years) baseline knowledge regarding antibiotics and antibiotic resistance was high although significant knowledge improvement was observed where baseline knowledge was low. Children's (5–11 years) knowledge around antibiotics and antibiotic resistance was significantly improved for all questions. The science show can be viewed as a success in improving parents' and children's knowledge of antibiotic use thereby highlighting the importance of educating the public through interaction.

## Introduction

Microbial resistance to antibiotics was first documented in 1947, just four years after they were mass-produced, and to this day it continues to be a growing problem for hospitals [1] and communities.^2^ There are numerous complex reasons for the increase in antibiotic resistance but it is clear that antibiotic use is associated with resistance [2]–[3]. The economic burden associated with multidrug-resistant bacteria in the EU is high [4], estimated to cause an economic loss of more than €1.5 billion each year [5]–[6]. Whilst we may never be able to completely eradicate antimicrobial resistance there have been numerous efforts to support interventions that encourage more appropriate use of antibiotics in an attempt to slow the progress of resistance [7].

The public play an important role in the reduction of antibiotic resistance bacteria [8]–[9]. However, recent studies show that there is still public demand for antibiotics [10] and misunderstanding about the activity of antibiotics against microbes and their prudent use [11]–[13]. This misunderstanding is reflected in the 2013 Eurobarometer survey, which found that although 84% of respondents were aware that taking too many antibiotics makes them ineffective, the main reason given by respondents for taking antibiotics is to treat flu or a cold, regardless of the fact that antibiotics do not kill viruses [14].

To tackle this public misunderstanding, resources have been invested in public and health care setting campaigns encouraging the responsible use of antibiotics [15]–[17], each with varying degrees of success. England has undertaken numerous campaigns to encourage the public to ask for fewer antibiotics [12] and make GPs more aware of their prescribing behaviour [18]–[19].

e-Bug, a junior and senior school educational programme, consists of eight teacher led lesson plans covering the spread, treatment and prevention of infection as well as basic information on microbes, both useful and harmful [20]. During 2007/08, in collaboration with the British Society for Antimicrobial Chemotherapy (BSAC), an e-Bug interactive science show was developed to take the key messages from the e-Bug school resource to the family environment. This science show covered microbes, hand hygiene, respiratory hygiene, food hygiene, antimicrobial resistance and prudent antibiotic use (Table 1). Due to the success of the e-Bug classroom activities in improving student knowledge [21], the science show interactive activities were modified versions of those in the e-Bug teacher's pack. This paper outlines the evaluation of the antibiotic awareness stand of this science show, examining its effectiveness of improving parent and child knowledge of antibiotic use and resistance.

**Table 1:** Overview of the science show stands.

Stand title	Activity description
Microbe Mania	Participants examine a series of microbial images and use these as a basis to make models of microbes in petri dishes. The demonstrator discusses their microbe of choice and provides more detailed information.
Horrid Hands	Using a fluorescent powder, participants observe how difficult it is to wash away unwanted microbes from your hands. A pepper and water activity shows why using soap to wash your hands is better than water alone.
Kitchen Mayhem	Participants make a chicken salad/sandwich from playdough and get an unexpected surprise when they see how far the microbes have spread across other food if they don't wash their hands and kitchen surfaces after cutting raw chicken.
Super Sneezes	A giant head and snot gun show participants how far their sneezes really travel and how a tissue reduces spread.
Antibiotic Awareness	An acid/base colour change titration demonstrates to participants the importance of finishing your course of antibiotics.

## Materials and Methods

### Study population

The science show evaluation was carried out over two separate weeks in June and August 2011, at a family holiday resort in England. The resort had a residential population of 8674 during week one in June and a residential population of 5222 during week two in August. The resort also has day visitors but we have no details of this population. The target group was families; parents (19+ years) and children (5–11 years). Science show demonstrators handed out flyers to families throughout the resort advertising the science show, flyers were also included in resident welcome packs therefore participants were self selected. Minimum sample size was conservatively determined as that required to detect a 10% difference between the before and after section percentage correct as statistically significant at the 5% level, 80% power and a baseline correct percentage of 80%, on the assumption that the before and after percentage correct are mutually independent.

### The intervention

The science show consisted of an initial 3 minute presentation, to all present, on microbes followed by a guided visit to 5 interactive stalls, covering the topics of microbes, hand hygiene, respiratory hygiene, food hygiene and antibiotic awareness (Table 1). Each stall comprised of a 3×2 m backing stand with background information, an interactive activity, and discussions with a trained demonstrator. Shows started every hour on the hour and lasted approximately 40 minutes with ten minutes before and ten minutes after each show for questionnaire completion.

### Questionnaire

Data were obtained by before and after knowledge change questionnaire. The questionnaire was piloted for readability and understanding, during a pilot study investigation at a different venue of the same holiday resorts during April 2009. Modifications to the questionnaire were made based on suggestions and lessons learned. The questionnaire was divided into five sections containing 26 statements covering the topics of microbes, hand hygiene, respiratory hygiene, food hygiene and antibiotic awareness; this paper will focus on the antibiotic awareness section which contained seven questions (Table 2). Statements relating to the learning outcomes of each stand were read aloud to each group and participants were asked to tick ‘true’, ‘false’ or ‘don't know’ for each statement. As participants were minors, verbal parental consent was obtained before giving children questionnaires. Questionnaires were only given to children whose parents have verbal consent. Parents stayed with their children throughout the completing the questionnaire process and during the science show. Demonstrators assisted in the completion of the questionnaires thus ensuring that ticked boxes were representative of participant responses consequently eliminating the possibility that parents could provide the children with the correct answers, or vice versa. Participant names were collected to allow matched before and after data comparisons. Questionnaires were collected in line with the data protection act 1998 and Caldicott 1999 regulations on handling and distributing sensitive participant information.

**Table 2:** Antibiotic awareness knowledge questions showing baseline percentage correct responses for children and adults.

Antibiotic Awareness Questions	Correct Response
Antibiotics:	
kill bacteria	True
kill viruses	False
Most coughs and colds get better without antibiotics	True
If you overuse antibiotics they are less likely to work in the future	True
Antibiotic resistant bacteria are caused by hospitals	False
You should keep any leftover antibiotics to treat infections in the future	False
Antibiotics also kill our good bacteria	True

### Ethical considerations

Previous consultation with the South West Multicentre Research Ethics Committee (MREC) confirmed that consent need not be sought from an NHS Research Ethics Committee (NHS REC) for this type of study as it did not involve NHS patients, staff or facilities [22]. This is in accordance with the National Research Ethics Service ‘defining research’ guidelines, which characterise the study as ‘service evaluation’ for the purposes of ethical review. These guidelines can be accessed at the following link: http://www.nres.nhs.uk/applications/is-your-project-research/. Provided anonymised results were published, no ethical review was needed as the study was educational and posed no potential harm for participants. As such, a waiver of approval was not required or sought from the South West Multicentre Research Ethics Committee [MREC].The study team did however retrospectively contact the Public Health England research and governance office who supplied us with a letter of exemption for the study. On completion of a questionnaire participating children were entered into a prize draw; there was no financial incentive to take part.

### Data analysis

Responses were coded and double entered into a customised EpiData 3.1 database by two separate individuals to ensure accuracy. Any question not answered by an individual was left blank in EpiData and this question was removed from any subsequent analysis for that individual. Discrepancies identified during double data entry were rectified by referring back to the hard copy questionnaire and ensuring that individual's response was entered accurately. The effectiveness of the science show in each age group was assessed by analysis of both individual and grouped statement data with M^c^Nemar's test for paired proportions and paired t- or sign test, as appropriate, respectively, with estimates of odds ratio, in the former, and percentage improvement, in both cases, also being calculated. Odds ratios close to one (together with their 95% confidence intervals (CI) spanning one), improvement scores close to zero (together with their 95% CI spanning zero) and p-values greater than 0.05 indicate no significant improvement.

## Results

**Figure 1:**
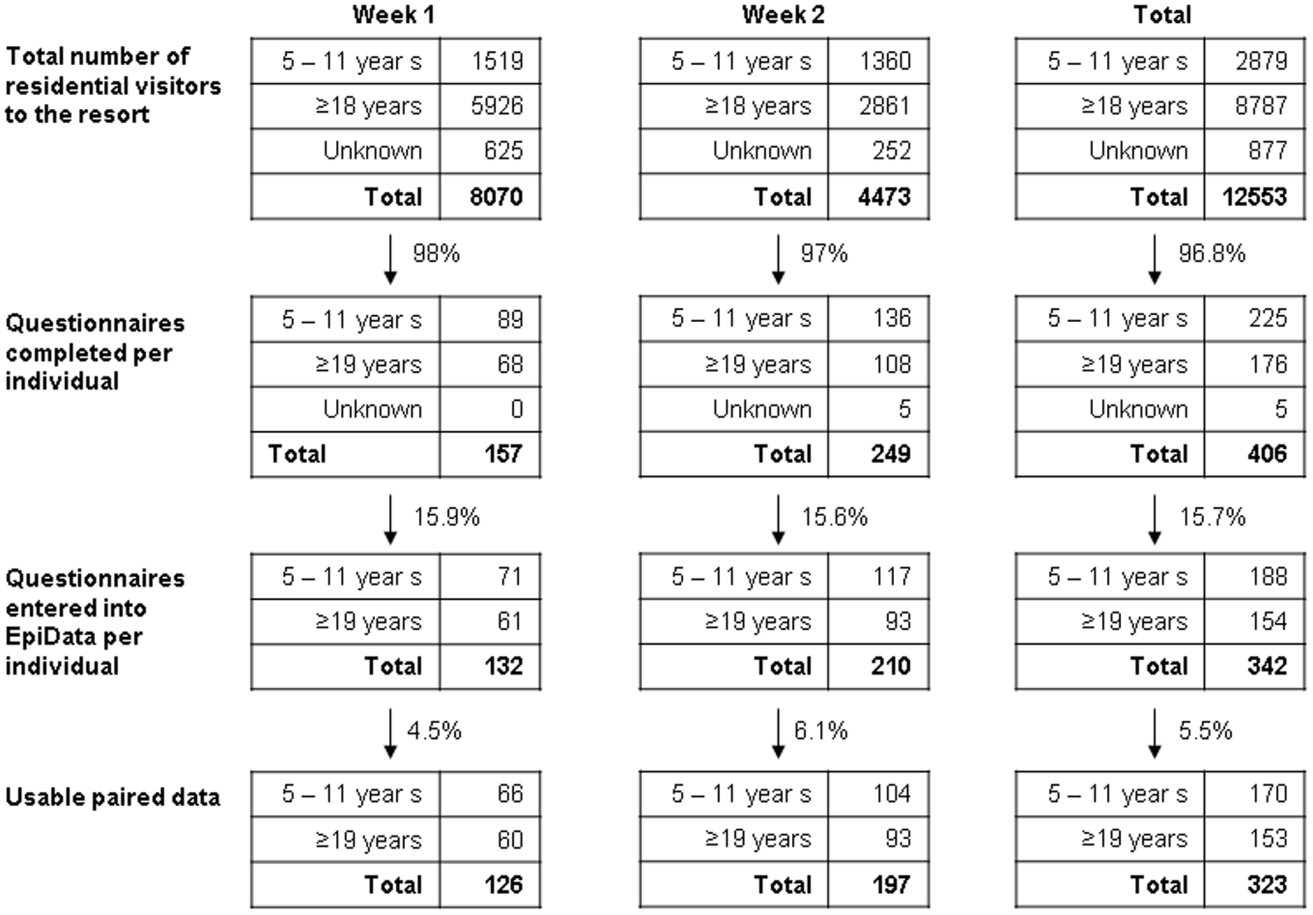
Participant recruitment.

Questionnaires were collected from 406 participants, but only 342 were entered for analysis (84.3%) after excluding responses from those that either failed to complete both the before and after section of the questionnaire, or where parents provided children with the correct answer or where participants were identified by parents as being autistic(Figure 1).

Sample size, over the two combined weeks, in the 5–11 year old and 19 years and above age groups was 188 and 154 respectively. However, failure to complete the antibiotic section in either the pre, post or both parts of the questionnaire resulted in final usable paired data sample sizes of 170 (5–11 years) and 153 (≥19 years). As such, the analysis described above was performed on these two age groups separately. A descriptive comparison of collected data between the two individual weeks did not reveal any substantial differences. Therefore, the data for both weeks were combined and no adjustment for week was made in the analysis presented in this paper.

### Knowledge improvement in children (Table 3)

**Table 3:** Improvement scores by statement for children [5–11 year old age group].

Question	% Correct at baseline [*n*]	% Improvement [95% CI]	odds ratio [95% CI]	*p* value
Antibiotics:				
kill bacteria	54.9 [169]	26.8 [17.4, 36.2]	5.0 [2.6, 10.6]	**<0.001**
kill viruses	30.9 [167]	30.9 [20.7, 41.1]	4.6 [2.5, 8.8]	**<0.001**
Most coughs and colds get better without antibiotics	53.5 [175]	11.8 [0.9, 22.6]	1.6 [1.0, 2.7]	**0.04**
If you overuse antibiotics they are less likely to work in the future	47.0 [169]	18.9 [8.9, 28.9]	2.7 [1.6, 5.0]	**<0.001**
Antibiotic resistant bacteria are caused by hospitals	30.5 [169]	25.0 [14.9, 35.1]	3.6 [2.0, 6.6]	**<0.001**
You should keep any leftover antibiotics to treat infections in the future	50.6 [173]	28.9 [19.2, 37.9]	5.4 [2.8, 11.3]	**<0.001**
Antibiotics also kill our good bacteria	39.9 [173]	23.8 [13.6, 34.1]	3.1 [1.8, 5.5]	**<0.001**

Children's knowledge in the 5–11 year old age group pre intervention for each of the seven questions ranged from 30.5%–54.9%. Overall, for this section of the questionnaire, less than half (42%) of the participants responded correctly at baseline. There was a marked improvement in antibiotic knowledge post intervention with an overall increase for all seven questions of 25% (*p*<0.001). Improvement was also observed for all individual statements with more than half the participants answering correctly for each question post intervention (55.5%–81.7%). The greatest percentage improvement (30.9%) was observed for the statement “*Antibiotics kill viruses*” with the smallest improvement for the statement “*Most coughs and colds get better without antibiotics*” (11.8%). Baseline knowledge however, was already quite high for this statement with over half the respondents (53.5%) answering correctly at baseline.

### Knowledge improvement in adults (Table 4)

**Table 4:** Improvement scores by statement for adults [≥19 year old age group].

Question	% Correct at baseline [*n*]	% Improvement [95% CI]	odds ratio [95% CI]	*p* value
Antibiotics:				
kill bacteria	64.9 [146]	24.3 [15.6, 33.1]	8.2 [3.2, 26.6]	**<0.001**
kill viruses	52.4 [145]	23.1 [14.4, 31.8]	7.8 [3.1, 25.4]	**<0.001**
Most coughs and colds get better without antibiotics	87.4 [151]	4.0 [−3.0, 11.0]	1.7 [0.7, 4.3]	0.3
If you overuse antibiotics they are less likely to work in the future	94.7 [151]	0.0 [−5.2, 5.2]	1.0 [0.3, 3.7]	>0.999
Antibiotic resistant bacteria are caused by hospitals	68.0 [147]	11.6 [2.8, 20.4]	2.5 [1.2, 5.7]	**0.01**
You should keep any leftover antibiotics to treat infections in the future	95.4 [152]	0.0 [−4.7, 4.7]	1.0 [0.2, 4.4]	>0.999
Antibiotics also kill our good bacteria	61.4 [153]	15.0 [5.9, 24.1]	2.9 [1.5, 6.2]	**0.001**

Baseline or pre intervention knowledge for adults, ≥19 years, was very high; an overall correct response rate of 75% at baseline with individual statement responses ranging from 52.4%–95.4%. Following the science show no significant knowledge change was observed for the statements *“Most coughs and colds get better without antibiotics”, “If you overuse antibiotics they are less likely to work in the future”* and *“you should keep left over antibiotics to treat infections in the future”* as correct baseline responses were extremely high; 87.4%, 94.7% and 95.4% respectively. For the remaining four statements however, a significant knowledge improvement was observed with percentage improvements for each question ranging from 11.6%–24.3%.

## Discussion

### Main findings

Children's baseline knowledge on antibiotics and their usage is quite low; a correct response for all questions was less than 55%. Knowledge following intervention was very high, ranging from 55.5% to 81.7% for all questions. Adult baseline knowledge regarding antibiotics was higher with correct response at baseline for questions ranging from 52.4% to 95.4%. Our findings show that pre intervention, 87.4% of adults correctly responded that many coughs and colds get better on their own. However, adults were least knowledgeable (52.4% correct response at baseline) on whether antibiotics could be used to treat viral infections.

### Other work in this area

The large improvement in children's knowledge may be attributed to the interactive and group learning experience which children prefer [21]. Children are the largest consumers of antibiotics but research has suggested that their knowledge of medicine is poor, despite the fact that they have more control in medicine use than most adults would predict [23]. As children are the antibiotic prescribers and users of the future it is essential that we continue to invest in their education thereby allowing them to make informed decisions in later life. Research carried out by Bush [24] indicates that children who had knowledge of medicine felt more in control of their own health. Individuals who are the most knowledgeable about antibiotics behave more responsibly [14]. To improve the use of antibiotics in childhood infections it is essential that parents be continuously reminded about prudent antibiotic use as they play a highly influential role in the medicine use of their children [23]. Newell found that children's misconceptions about antibiotics start at an early age and may be influenced by their parents health seeking behaviour and expectations for antibiotics [25]. Therefore, perhaps future studies should aim to observe any correlation between parent and child knowledge and/or attitude towards antibiotic use.

Our findings suggest that adults questioned are aware of the importance of not storing or using leftover antibiotics however, a recent omnibus survey found an increase in the percentage of people retaining leftover antibiotics in England between 2008 and 2009 [12]. Emslie and Bond [26] surmised that although the public are aware of the dangers of antibiotic misuse to their own health, their behaviour and practice don't always reflect this awareness. This may be because parental beliefs, fears and expectations play an important part in consulting behaviour suggesting that as well as increasing knowledge about antibiotic use and resistance we need to increase adults and parents confidence to self care for the majority of infections that are self limiting without consulting their general practitioners.

2010 Eurobarometer findings demonstrate that individuals who are exposed to messages on prudent antibiotic use have absorbed that information and are more likely to change their usage habits [27]. Previous research carried out in the US has also noted that individuals have a higher awareness of prudent antibiotic use when exposed to 2 or more interventions [28]. Whilst mass media campaigns are known to increase awareness and knowledge and reinforce existing attitudes [29], it has been suggested that interpersonal channels, such as the interactive science show with a trained demonstrator, work better at changing attitudes and behaviour [30]. Indeed, Huttner *et al.* [17] concluded that multifaceted campaigns repeated over several years have greatest effect. Taking this into consideration, it may be concluded that for campaigns to be truly successful, a focussed multi channel approach combining mass media and interpersonal channels be adopted.

Public health organisations in England have undertaken various campaigns to encourage the public to ask for fewer antibiotics however there is little evidence that the traditional poster based campaigns used in isolation were effective [12]. The 2010 Eurobarometer report concluded that messages on antibiotic use obtained from doctors was more effective than the media [27], suggesting that the power of persuasion is considerably stronger than the media. Whilst this may have merit, we would like to elaborate further by suggesting that the public benefit from the interactive approach where they can address their individual concerns and relate these to the key antibiotic usage messages, whether that is in a GP surgery or an educated demonstrator at a science show. It is also evident that the greatest predictor of antibiotic use is previous antibiotic use and consultation rate [27] suggesting that if we can increase the importance of prudent and appropriate antibiotic use to the general public we may be able to reduce their consultation rate but we also need to increase their confidence in self care.

### Strengths and Weaknesses

The science show activities were carried over two separate weeks during the summer months using two different groups of trained demonstrators thus mimicking how the science show is carried out when newly trained individuals use the resources in different settings with the general public. The intervention took place in a family holiday resort resulting in participants being from a large demographic catchment. Our questionnaire response rate for those attending the science shows was very high at 73% therefore we believe our results are robust. The science show activities are based on the successful e-Bug project thereby providing united key messages to parents and children and reinforcing school learning outside the classroom.

Whilst participation was based on self selection rather than random selection we consider this approach to be generalisable to other science show settings where participants also self select. Failure to obtain a knowledge retention questionnaire from participants' means we have no information as to whether or not knowledge change via the intervention has lasting effects, however our previous work with the younger age group suggests that knowledge improvement gained via interactive activities is lasting [21].

### Implications

Overall the e-Bug science show can be viewed as a success in improving parents and children's knowledge of antibiotic use thereby highlighting the importance of educating the public through interaction. As such, the use of a science show like e-Bug should be encouraged in various educational settings to help promote prudent antibiotic use. Currently the science show visits science festivals and family holiday resorts and as such, targets a fairly narrow population. In order to increase awareness of prudent antibiotic use to the wider general public and based on the fact that heightened awareness is achieved when exposed to two or more interventions [28], we should consider extending/implementing the reach of the science show so that it can be used to reinforce similar key messages from other interventions. This may be achieved by making the materials available to other professional or voluntary organisations who participate in public or school outreach programmes e.g. environmental health departments, universities, charities and local councils. The backing stand posters could also be made available to GP surgeries, pharmacists or hospital waiting rooms and foyers.

### Future work needed

Knowledge improvement does not necessarily change attitude or behaviour. The inclusion of an interactive take home booklet on common self-limiting illnesses [31] to the science show resources may improve public confidence to self-care and thus reduce GP consultation rates for these self-limiting illnesses. The science show resources make up one arm of e-Bug, a project striving to achieve a focussed multi faceted approach through: interpersonal channels; educational resources for schools [and the science shows], and through the mass media route: forging links with international campaigns such as European Antibiotic Awareness Day and Global Hand Washing Day. The recent development of an online interactive version of the science show, in collaboration with BSAC, will facilitate further learning for families who have attended the event and wider reach of the science show to those who cannot attend an event. The e-Bug initiatives allow us to communicate familiar and consistent prudent antibiotic awareness messages to the general public.
